# Mutant huntingtin confers cell-autonomous phenotypes on Huntington’s disease iPSC-derived microglia

**DOI:** 10.1038/s41598-023-46852-z

**Published:** 2023-11-22

**Authors:** Nina Stöberl, Jasmine Donaldson, Caroline S. Binda, Branduff McAllister, Hazel Hall-Roberts, Lesley Jones, Thomas H. Massey, Nicholas D. Allen

**Affiliations:** 1 School of Biosciences, Cardiff University, Cardiff, UK; 2 Centre for Neuropsychiatric Genetics and Genomics, Division of Psychological Medicine and Clinical Neurosciences, Cardiff University, Cardiff, UK; 3 UK Dementia Research Institute at Cardiff, Cardiff University, Cardiff, UK

**Keywords:** Neuroimmunology, Microglia

## Abstract

Huntington’s disease (HD) is a neurodegenerative disorder caused by a dominantly inherited CAG repeat expansion in the huntingtin gene (*HTT*). Neuroinflammation and microglia have been implicated in HD pathology, however it has been unclear if mutant *HTT* (m*HTT*) expression has an adverse cell-autonomous effect on microglial function, or if they are only activated in response to the neurodegenerative brain environment in HD. To establish a human cell model of HD microglia function, we generated isogenic controls for HD patient-derived induced pluripotent stem cells (iPSC) with 109 CAG repeats (Q109). Q109 and isogenic Q22 iPSC, as well as non-isogenic Q60 and Q33 iPSC lines, were differentiated to iPSC-microglia. Our study supports a model of basal microglia dysfunction in HD leading to elevated pro-inflammatory cytokine production together with impaired phagocytosis and endocytosis capacity, in the absence of immune stimulation. These findings are consistent with early microglia activation observed in pre-manifest patients and indicate that m*HTT* gene expression affects microglia function in a cell-autonomous way.

## Introduction

Huntington’s disease (HD) is a severe neurodegenerative disorder caused by a dominantly inherited CAG trinucleotide repeat expansion in the huntingtin gene (*HTT*) on chromosome 4, resulting in progressive motor, psychiatric and cognitive problems that culminate in dementia and death^1^. HD is the most common polyglutamine expansion disease, with a prevalence of around 10.6–13.7 individuals per 100,000 in Western populations^2^. The huntingtin protein is ubiquitously expressed, with high expression in the brain^3^. Although it is expressed throughout the brain in different neuronal subtypes, brain regions show different vulnerabilities to degeneration, with medium spiny neurons of the striatum being most severely affected^4^.

Several PET studies have demonstrated an involvement of neuroinflammation in HD, as microglia activation correlates with disease severity and pathology in HD patients^5,6^. Microglia are the principal resident innate immune cells of the central nervous system (CNS)^7^ and their activation is evident in presymptomatic HD gene carriers, being detectable up to 15 years before predicted age of disease onset^8^. PET findings have been confirmed by autopsy studies where activated microglia have been found in the most prominent regions that are affected in HD (striatum and cortex), in all stages of HD pathology. Furthermore, increased accumulation of activated microglia correlated with increased disease severity and neuronal loss in affected regions^9^. Despite the observations of early microglial activation, the contribution of pre-symptomatic neuroinflammation to disease onset and progression is poorly understood.

To model early microglia activation in HD we used human patient-derived induced pluripotent stem cells (iPSC). In recent years, many iPSC-based human HD cell models have been generated and used to study neuronal and astrocyte phenotypes in HD (summarized by^10^). The use of isogenic control lines enables the effects of the *HTT* CAG repeat expansion to be studied without confounding factors introduced by different genetic backgrounds. A number of isogenic HD-iPSC/ESC lines have been generated, and various mutation-related phenotypes have been reported to be rescued by genetic correction of the HD-mutation to a non-pathogenic repeat length. These include phenotypes related to mitochondrial dysfunction, neural rosette formation, disrupted neurogenesis, sensitivity to stressors, neuronal viability, DNA damage and gene expression changes^11–15^.

The Q109 iPSC are amongst the most studied patient-derived lines in use for modeling HD. They respond well to multiple neural and glial differentiation protocols and in contrast to some other lines exhibit the hallmark feature of somatic CAG repeat expansion. Here we have used CRISPR-Cas9 assisted homologous recombination to integrate a piggyBac transposon vector to generate foot-print free isogenic HD iPSC of the Q109 iPSC, which were previously generated from fibroblasts of an individual with childhood-onset HD^16,17^. The pathogenic repeat length was originally 109 *HTT* CAG repeats but has increased over time in culture to ~ 120 CAG repeats.

Previous studies have provided evidence that HD-related differences in microglial function include cell-autonomous effects of m*HTT* expression, rather than merely being a secondary consequence of neuronal pathology^18,19^. For example, expression of m*HTT* in immortalized mouse microglial BV2 cells and HD mice upregulated Spi1 regulated pro-inflammatory gene expression^18^. Furthermore, HD microglia, derived from human embryonic stem cells (ESC), were hyper-reactive to pro-inflammatory stimulation in the production of pro-inflammatory cytokines, released elevated levels of reactive oxygen species, showed increased levels of apoptosis and were more susceptible to exogenous stress^19^. Here, we further characterized microglia morphology and functional phenotypes in HD and isogenic control iPSC lines. We provide the first evidence of m*HTT*-induced changes to basal pro-inflammatory cytokine production in microglia, in the absence of immune stimulation. We also found that the endocytic and phagocytic capabilities of m*HTT*-bearing microglia were reduced under basal conditions, suggesting a potential role of microglial cell-autonomous inflammation and activity in the earliest phases of HD. In contrast to basal activation status, HD and control microglia showed similar responses to pro-inflammatory stimulation.

## Results

### Genetic correction of HD Q109 iPSC

**Figure 1:**
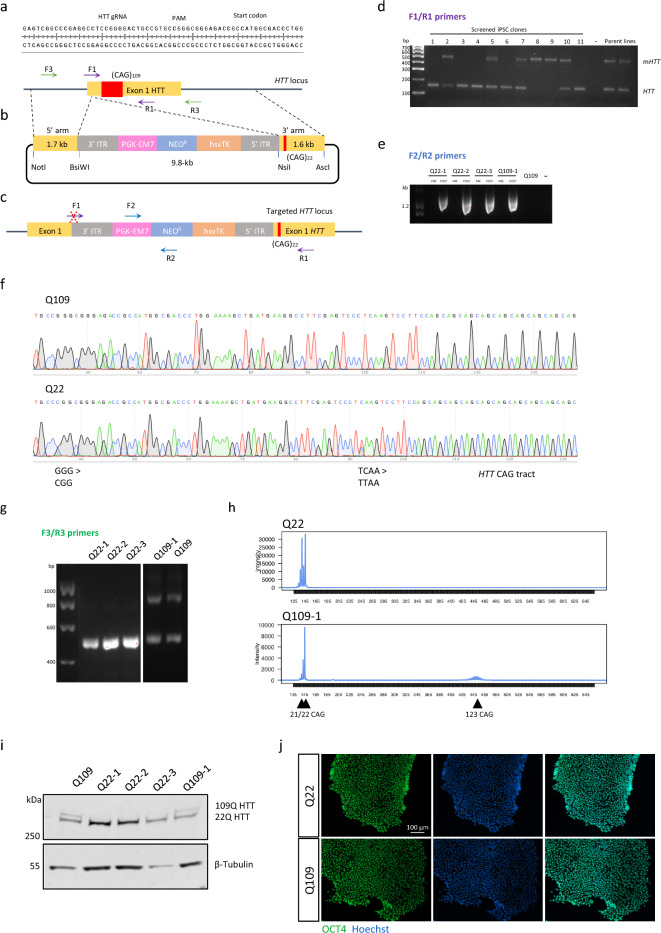
Design and generation of isogenic Q22 iPSC using piggyBac and CRISPR-Cas9 mediated homologous recombination. (**a**) Schematic showing *HTT* sequence and CRISPR-Cas9 guide RNA target site. (**b**) Schematic depicting the donor DNA and piggyBac transposon-based selection strategy used to target the CAG tract in exon 1 of the *HTT* locus. The 5′ and 3′ homology arms of the plasmid are 1.7 kb and 1.6 kb, respectively. (**c**) Schematic of targeted *HTT* locus and indication of forward and reverse (F/R) primers used for genotyping in panels (**d**) and (**e**). (**d**) Diagnostic PCR screen amplifying exon 1 of *HTT* locus using F1/R1 primers. Insertion of the targeting repair vector disrupted the forward primer binding site, and thus no PCR amplification was detected in the alleles that were successfully targeted. PCR identified clones in which the WT allele or the mutant allele had been targeted. (**e**) PCR screen amplifying the selection cassette illustrates loss of selection cassette in selected clones after excision. (**f**) Sanger sequencing of exon 1 of the *HTT* locus using F3/R3 primers confirmed excision of the piggyBac cassette from TTAA integration sites and introduction of silent PAM site mutations (GGG > CCG). (**g**) PCR amplifying exon 1 of the *HTT* locus in selected clones verified successful correction at the *HTT* locus. (**h**) Electropherograms of fluorescent PCR across the CAG repeat showed two WT alleles (21 CAG, 22 CAG) present and loss of the expanded allele in Q22 iPSC. Clone Q109-1 retained the expanded repeat, and the WT allele is re-inserted with a CAG repeat length + 1 of original WT allele. (**i**) Western blot showing expression of wildtype size HTT (22Q) following cassette excision, confirming successful correction at the *HTT* locus in selected clones. (**j**) Gene-corrected clones maintained expression of the pluripotency marker OCT4, shown by immunostaining. Scale bar = 100 μm. Uncropped gels and plots are presented in Supplementary Fig. S5.

To correct the disease mutation at the *HTT* locus in HD Q109 iPSC we used CRISPR/Cas9 and homology-directed repair using a construct with a piggyBac transposon selection cassette^20^. Homologous recombination was facilitated by making a double-stranded DNA break upstream of exon 1 using CRISPR/Cas9 and a previously validated single guide RNA (sgRNA; An et al. 2012) (Fig. 1a). The piggyBac vector contained 1.7 Kb and 1.6 Kb 5′ and 3′ homology arms flanking a 22 CAG repeat, so that homologous recombination of the vector would replace the expanded CAG repeat tract (originally 109 CAGs) with a wild-type (WT) repeat tract (22 CAGs) (Fig. 1b). To enable piggyBac vector transposase excision, a silent mutation was introduced into the left homology arm, converting a TCAA tetra-nucleotide to TTAA 61 bp 3′ to the double strand break site. In addition, a silent mutation was introduced to destroy the sgRNA PAM site (CGG > CCG). The piggyBac vector contained both a neomycin-resistance gene for positive selection of vector integration, and the herpes simplex virus thymidine kinase gene for negative selection for subsequent piggyBac vector sequence removal (Fig. 1b).

Following nucleofection and selection of Q109 iPSC, 288 colonies were screened by PCR amplification across the *HTT* CAG repeat (Fig. 1c,d). Targeted candidates showed PCR amplification of the 22 CAG repeat allele in the absence of the expanded allele product, as vector integration interrupted the forward primer sequence (Fig. 1c). Of the 288 colonies screened, targeting of the expanded allele occurred in five clones (1.7%) and targeting of the wild-type allele occurred in three clones (1.0%).

To remove the selection cassette, targeted Q109 iPSC were transfected with the piggyBac excision-only vector, followed by negative selection with 1 µM ganciclovir. Resistant colonies were screened by PCR to confirm that clones were free of the piggyBac selection cassette at the *HTT* locus and elsewhere in the genome where the cassette may have randomly integrated (Fig. 1e). Sanger sequencing confirmed successful introduction of the silent mutations TCAA > TTAA and GGG > CGG in the targeted alleles and effective excision of the selection cassette (Fig. 1f and Supplementary Fig. S1a). The absence of the expanded CAG repeat tract was demonstrated by Sanger sequencing (Supplementary Fig. S1), gel electrophoresis (Fig. 1g) and capillary electrophoresis (Fig. 1h). Western Blot confirmed expression of wild-type HTT and absence of mutant HTT in Q22 clones following cassette excision (Fig. 1i).

Three Q109-corrected clones were taken forward for further characterization: Q22-1, Q22-2, Q22-3, as well as one Q109 iPSC clone (Q109-1) where the WT allele (21 CAGs) had been targeted and was re-inserted with a CAG repeat length + 1 CAG. This provided a control cell line with 22/109 CAG repeats that had undergone the targeting process. iPSC clones maintained their stem-cell characteristics as evidenced by robust OCT4 staining (Fig. 1j).

Unbiased detection of potential sgRNA/CRISPR off-target sites was performed using the software system CRISPOR^21^ and the top 10 off-target regions were assessed with Sanger sequencing (Supplementary Table S1). There were no off-target indels at these loci in any of the isogenic clones.

Whole-exome sequencing was also performed on genomic DNA from Q109 and all isogenic corrected clones to characterize their exonic background, and to determine whether significant mutations had occurred through general cell culture maintenance. Considering variants with a mean read depth of at least 10, 467 variants were found to differ between cell-lines. Most of these variants (370/467; 79.2%) were synonymous and presumed to be of no functional consequence. The remaining 97 were non-synonymous coding changes (Supplementary Table S2). Reassuringly, these variants were not in known HD or microglia relevant genes. Instead, many appeared to be involved in signaling or growth pathways. It is well established that prolonged iPSC culture, especially under selective pressures, can lead to adaptations such as increased growth rate or reduced apoptosis^22^. To assess any chromosomal abnormalities, virtual karyotyping was performed. We did not detect any duplications or deletions in the corrected clones that were not already present in the parent clones (Supplementary Table S3).

### Q109 iPSC differentiate to iPSC-microglia but show impaired attachment to fibronectin and increased basal levels of cytokine secretion

Three individual clones of the iPSC lines Q22 (Q22-1, Q22-2, Q22-3; collectively called Q22) and Q109 (Q109n1, Q109n4, Q109n5; collectively called Q109), respectively, were differentiated to microglia precursor cells (MPC) following an established protocol^23^. Q22 and Q109 iPSC showed no differences in differentiation, enabling free-floating MPC that were immunopositive for CD11b, CD14, and CD45 to be harvested weekly for analysis from culture supernatants over a period of 4–8 weeks (Supplementary Fig. S2).

**Figure 2:**
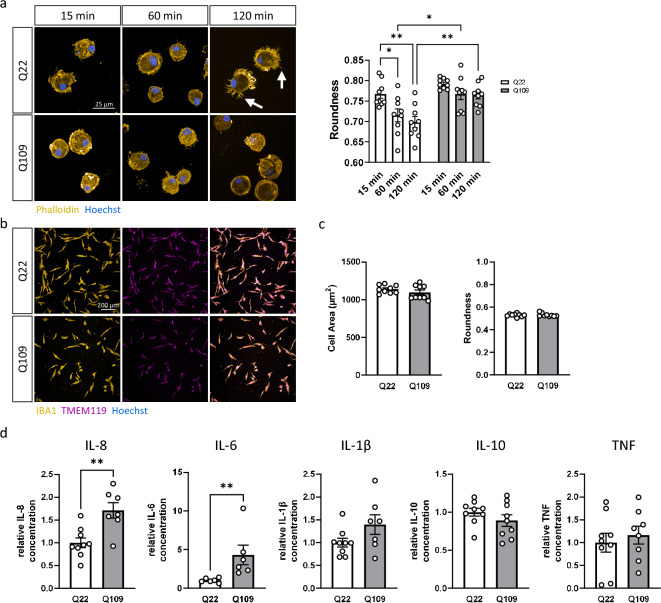
Q109 iPSC differentiated to iPSC-microglia and showed no morphological differences to Q22 control iPSC-microglia but exhibit impaired attachment to extracellular matrix and increased cytokine secretion. (**a**) Investigation of MPC attachment to the extracellular matrix protein fibronectin 15 min, 60 min and 120 min after plating using phalloidin staining. Arrows indicate finely branched filopodia like protrusions. Roundness of Q22 MPC decreased significantly over time. This decrease was not detected in the Q109 MPC. There is no difference in roundness between Q109 and Q22 MPC 15 min after plating, but at 60 min and 120 min. Scale bar = 25 μm. (**b**) Confirmation of cell identity of both Q22 and Q109 iPSC-microglia using IBA1 and TMEM119. Scale bar = 100 μm. (**c**) Investigation of iPSC-microglia morphology showed no differences between Q22 and Q109 iPSC-microglia in their cell area and roundness. (**d**) Unstimulated iPSC-microglia supernatants were assessed by flow cytometric analysis for the secretion of the cytokines IL-8, IL-6, IL-1β, IL-10, TNF and IL-12p70. IL-12p70 secretion was below detection level. Unstimulated Q109 iPSC-microglia showed an increased secretion of IL-8 and IL-6. No difference was detected for the secretion of IL-1β, IL-10 and TNF. Data are expressed as mean ± SEM. n = 3 independent experimental repeats with 2–3 clones. Statistical analysis was performed using two-way ANOVA and Tukey’s honest significance test or unpaired t-test and Mann–Whitney *U* test (for IL-6). *p < 0.05; **p < 0.01. min = minutes.

For terminal differentiation to a microglia phenotype, floating MPC were plated onto fibronectin, a glycoprotein of the extracellular matrix, and cell spreading monitored for 120 min. Q109 MPC showed an impairment in their interaction with fibronectin seen as a delay in attachment and spreading after plating. While Q22 MPC quickly started to spread and ramify on the matrix, displaying finely branched filopodia-like protrusions (see white arrows, Fig. 2a), Q109 MPC exhibited no change in roundness 2 h after plating (Fig. 2a). Q109 MPC did however attach after overnight culture (not shown), and after 2 weeks of terminal differentiation, supported by astrocyte conditioned medium^24^, both Q109 and isogenic Q22 iPSC-microglia expressed the cell-type specific markers ionized calcium-binding adapter molecule 1 (IBA1) and transmembrane protein 119 (TMEM119) (Fig. 2b). Image analysis of the F-actin cytoskeleton in Q22 and Q109 iPSC-microglia, stained with phalloidin, showed no differences in their morphology, as quantified by cell area and roundness, at terminal differentiation (Fig. 2c). Taken together, the results suggest that, besides the delayed attachment and cell spreading, Q109 iPSC are not compromised in microglial differentiation.

Microglia are major mediators of inflammation in the brain. They respond to changes in their environment by producing cytokines, chemokines and by upregulating immunomodulatory surface markers^25^. Cytokines are used to communicate to other cells and are released for auto- and paracrine signaling^26^. To best model microglial activity relevant to early premanifest stages of HD Q109 and Q22 iPSC-microglia were assessed for the secretion of cytokines under basal conditions, without pro-inflammatory stimulation. The BD Human Inflammatory Cytokine Kit allowed for the detection of IL-8, IL-6, IL-1β, IL-10, IL-12p70 and TNF in the iPSC-microglia culture supernatant via flow cytometric analysis. Q109 and Q22 iPSC-microglia secretion of IL-12p70 was below the detection threshold of the kit. Secretion of the pro-inflammatory cytokines IL-8 was detected to be significantly increased by 1.7-fold (*p* = 0.0024), and IL-6 by 4.3-fold (*p* = 0.0022), compared to the Q22 isogenic controls. No significant differences were detected for the cytokines IL-1β, IL-10 and TNF (Fig. 2d). These data suggest that Q109 iPSC-microglia have elevated basal pro-inflammatory cytokine production.

### Q109 iPSC-microglia show impaired phagocytosis and endocytosis and decreased numbers of late endocytic vesicles

**Figure 3:**
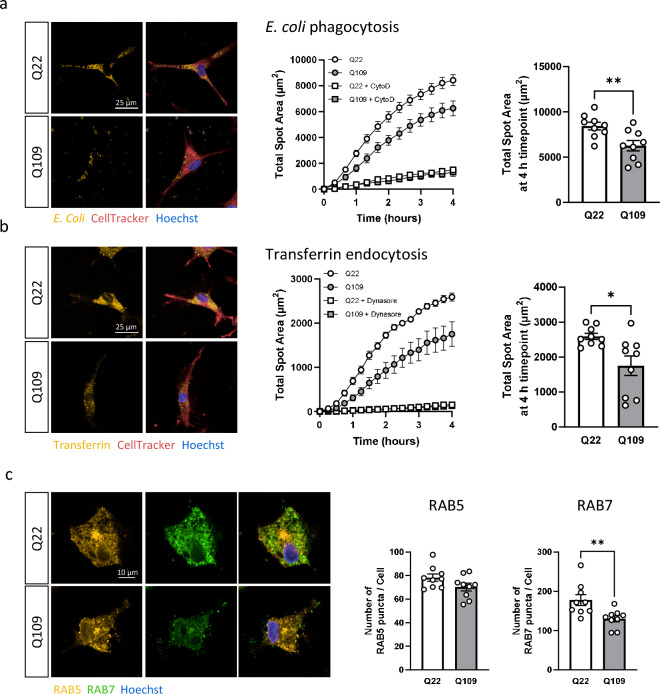
Q109 iPSC-microglia showed impairment in phagocytosis and endocytosis. (**a**) Phagocytosis was assessed via the uptake of pHrodo-conjugated *E. coli* particles. Q109 and Q22 iPSC-microglia were imaged every 20 min for 4 h. Uptake of *E. coli* particles was inhibited by the addition of 30 μM cytochalasin D (CytoD) for 1 h prior and during phagocytosis. Total spot area of phagocytosed *E. coli* after 4 h was significantly reduced in Q109 iPSC-microglia compared to Q22 control iPSC-microglia. (**b**) Endocytosis was assessed via the uptake of pHrodo-conjugated transferrin particles. Q109 and Q22 iPSC-microglia were imaged every 15 min for 4 h. Uptake of transferrin particles was inhibited by the addition of 30 μM dynasore for 1 h prior and during endocytosis. Total spot area of transferrin particles was significantly reduced in Q109 iPSC-microglia compared to Q22 control iPSC-microglia. (**c**) Early and late endosomes were identified by immunostaining using the early endosome marker RAB5 and late endosome marker RAB7. Number of RAB7, but not RAB5 puncta/cell were significantly decreased in Q109 iPSC-microglia cells. Data are expressed as mean ± SEM. n = 3 independent experimental repeats with 3 clones. Statistical analysis was performed using unpaired t-test. *p < 0.05; **p < 0.01.

Microglia contribute to brain homeostasis by clearing of pathogens, apoptotic cells, and debris from the extracellular space by phagocytosis^27^. Q109 and Q22 iPSC-microglia phagocytosis was investigated by measuring the uptake of pHrodo-labelled *E. coli*. Addition of cytochalasin D (CytoD) inhibited phagocytic uptake via inhibition of actin cytoskeleton remodeling and was used as a negative control. Q109 iPSC-microglia showed significantly reduced phagocytosis of pHrodo *E. coli* particles compared to the Q22 isogenic control iPSC-microglia (*p* = 0.0076, Fig. 3a).

While phagocytosis is used to uptake foreign particles in large vesicles that can be greater than 500 nm in diameter, microglia can also take up smaller extracellular material via endocytosis^28^. Q109 and Q22 iPSC-microglia endocytosis was investigated by the uptake of pHrodo-labelled transferrin. As a negative control, addition of dynasore, a dynamin GTPase inhibitor, was used to inhibit endocytic uptake by disrupting membrane fission during endocytosis. Q109 iPSC-microglia showed a significantly decreased total uptake of pHrodo-labelled transferrin compared to the Q22 control iPSC-microglia (*p* = 0.0112, Fig. 3b). In addition to *E. coli* and transferrin, phagocytosis of pHrodo-labelled zymosan and endocytosis of pHrodo-labelled dextran (10 000 Da molecular weight) were assessed. No significant differences were detected in the uptake of these cargos (Supplementary Fig. S3), indicating that m*HTT* induced impairments in microglia phagocytosis and endocytosis are cargo-specific.

Particles internalized via endocytosis are sorted within the endocytic system and are transported via early and late endosomes to the lysosomes^29^. Commonly used markers for early and late endosomes are the small GTPases RAB5 and RAB7, respectively, which organize effector proteins into specific membrane subdomains^30^. Q109 and Q22 iPSC-microglia were co-stained for RAB5 and RAB7, and the number of RAB5- and RAB7-positive puncta investigated. Q109 iPSC-microglia showed significantly fewer RAB7- (*p* = 0.0086), but not RAB5-positive puncta (Fig. 3c).

Other assays run as part of the investigation of iPSC-microglia function included the measure of calcium responses to the calcium ionophore ionomycin, as well as the purines ATP and ADP. While ionomycin facilitates the transport of extracellular Ca^2+^ across the plasma membrane and releases Ca^2+^ from its intracellular stores, ATP and ADP trigger Ca^2+^ release via purinergic receptors with different specificities at the microglia membrane^31^. A significantly reduced calcium response to ionomycin (*p* < 0.0001) and ATP (*p* = 0.0013), but not ADP was detected for Q109 iPSC-microglia, compared with Q22 iPSC-microglia (Supplementary Fig. S4). Further, Q109 iPSC-microglia showed no defects in mitochondrial respiration, measured by the Cell Mito Stress Test^32^, or lysosomal protease activity, investigated by hydrolysis of DQ-BSA (Supplementary Fig. S4).

### Pro-inflammatory stimulation impairs Q109 and Q22 iPSC-microglia function equally

**Figure 4:**
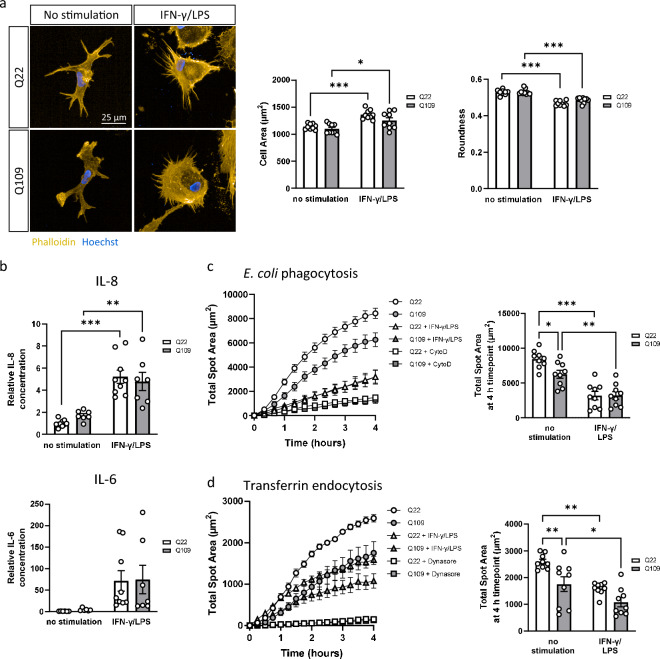
Effect of pro-inflammatory stimulation on Q22 and Q109 iPSC-microglia. (**a**) iPSC-microglia were stained for phalloidin and morphology was assessed. Both Q109 and Q22 pro-inflammatory stimulated iPSC-microglia exhibited a significantly increased cell area and decreased roundness compared to unstimulated cells. No differences were detected between Q109 and Q22 iPSC-microglia. (**b**) Pro-inflammatory stimulation increased secretion of IL-8 significantly, and IL-6 in both Q109 and Q22 iPSC-microglia. (**c**) Phagocytosis of *E. coli* particles was significantly reduced after pro-inflammatory stimulation in both Q109 and Q22 iPSC-microglia. The significant difference in phagocytosis capacity in unstimulated iPSC-microglia disappeared after pro-inflammatory stimulation. (**d**) Endocytosis of transferrin is significantly reduced after pro-inflammatory stimulation in both, Q109 and Q22 iPSC-microglia. The significant difference in endocytosis capacity in unstimulated iPSC-microglia disappeared after pro-inflammatory stimulation. Data are expressed as mean ± SEM. n = 3 independent experimental repeats with 3 clones. Statistical analysis was performed using two-way ANOVA and Tukey’s honest significance test. *p < 0.05; **p < 0.01; ***p < 0.001.

Microglia in the healthy mature brain have a ramified morphology associated with a resting state. Loss of brain homeostasis due to disease or trauma to the brain induces profound changes to microglia cell shape, such that they exhibit an amoeboid phenotype^33^. In iPSC-microglia, a pro-inflammatory state is experimentally induced by stimulation with LPS and/or IFN-γ^34^. Incubation with 20 ng/mL IFN-γ and 20 ng/mL LPS for 24 h induced loss of large, long-ranging ramifications, which were replaced with many fine and short ramifications in both Q109 and Q22 iPSC-microglia. Due to the fine ramifications, analysis of cell morphology showed an increased cell area (*p* = 0.0005 for Q22, *p* = 0.0192 for Q109) and decreased roundness (*p* < 0.0001 for Q22 and Q109) after stimulation (Fig. 4a). For both Q109 and Q22 iPSC-microglia, pro-inflammatory stimulation induced a significant upregulation in secreted IL-8 (*p* < 0.0001 for Q22, *p* = 0.0021 for Q109). Due to high variability, increased levels of stimulated IL-6 secretion did not reach significance. Stimulated IL-8 secretion was similar when comparing Q109 to Q22 iPSC-microglia, therefore the elevated basal secretion of IL-8 in Q109 iPSC-microglia did not translate to a hyperexcitable stimulated response (Fig. 4b).

Pro-inflammatory stimulation also significantly reduced phagocytosis of *E. coli* (*p* < 0.0001 for Q22, *p* = 0.0017 for Q109) and endocytosis of transferrin (*p* = 0.0016 for Q22, *p* = 0.0474 for Q109), in both Q109 and Q22 iPSC-microglia (Fig. 4c/d). The strong inhibitory effect of pro-inflammatory stimulation on phagocytosis and endocytosis eliminated (significant) differences between the Q109 and Q22 iPSC-microglia, as both were equally affected.

### HD iPSC-microglia with shorter HTT CAG repeat lengths show less severe phenotypes

**Figure 5:**
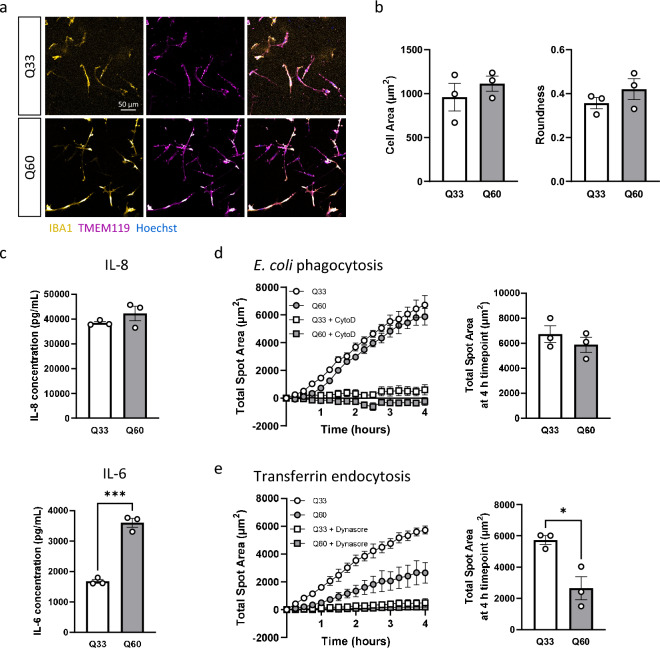
Q60 iPSC-microglia showed impairment in endocytosis and increased cytokine secretion compared to Q33 iPSC-microglia. (**a**) Confirmation of cell identity of both Q33 and Q60 iPSC-microglia using IBA1 and TMEM119. Scale bar = 50 μm. (**b**) Investigation of iPSC-microglia morphology showed no differences between Q33 and Q60 iPSC-microglia in cell area and roundness. (**c**) Unstimulated iPSC-microglia supernatants were assessed by ELISA for the secretion of the cytokines IL-8 and IL-6. Unstimulated Q60 iPSC-microglia showed an increased secretion of IL-6, but not IL-8. (**d**) Phagocytosis was assessed via the uptake of pHrodo-conjugated *E. coli* particles. Q33 and Q60 iPSC-microglia were imaged every 15 min for 4 h. Uptake of *E. coli* particles was inhibited by the addition of 30 μM cytochalasin D (CytoD) for 1 h prior and during phagocytosis. Total spot area of phagocytosed *E. coli* after 4 h was not different between Q33 and Q60 iPSC-microglia. (**e**) Endocytosis was assessed via the uptake of pHrodo-conjugated transferrin particles. Q33 and Q60 iPSC-microglia were imaged every 10 min for 4 h. Uptake of transferrin particles was inhibited by the addition of 30 μM dynasore for 1 h prior and during endocytosis. Total spot area of transferrin particles was significantly reduced in Q60 iPSC-microglia compared to Q33 control iPSC-microglia. Data are expressed as mean ± SEM. n = 3 independent experimental repeats with 1 clone. Statistical analysis was performed using unpaired t-test. *p < 0.05; ***p < 0.001.

In order to test if HD iPSC-microglia with a shorter *HTT* CAG repeat mutations show a similar phenotype to the Q109 iPSC lines, a second set of HTT/control iPSC was used. Q60 and Q33 iPSC lines are not isogenic but were derived from a pair of siblings, and were previously generated together with the Q109 iPSC lines^16,35^. Q60 and Q33 iPSC successfully differentiated to iPSC-microglia (Fig. 5a, Supplementary Fig. S2). Investigation of morphology showed no differences in cell area and roundness, similar to the Q109/Q22 comparison (Fig. 5b). The basal level of IL-6 secretion (*p* = 0.0003), but not IL-8, was significantly increased in Q60 compared to Q33 iPSC-microglia (Fig. 5c). Despite significantly decreased phagocytosis capacity in Q109 iPSC-microglia, Q60 iPSC-microglia showed no difference in the uptake of pHrodo labelled *E. coli* compared to Q33 iPSC-microglia (Fig. 5d). In contrast, transferrin endocytosis was significantly impaired in Q60 iPSC-microglia, consistent with the endocytosis impairment in Q109 iPSC-microglia (*p* = 0.0178, Fig. 5e). These findings indicate that cells expressing m*HTT*, but with shorter CAG repeat lengths, also affect microglia cytokine secretion and functional phenotypes, however with reduced severity.

## Discussion

Here, we described the genetic correction of HD iPSC from a CAG repeat length of > 109 to a WT repeat length of 22 using a CRISPR-Cas9 and piggyBac transposon-based homologous recombination approach. The Q109 line has been used extensively to investigate novel HD pathologies and to study regulation of CAG repeat expansion in vitro^36–40^, and therefore an isogenic control line with a wild-type *HTT* CAG repeat length would be a valuable tool for probing HD-relevant pathology. Investigating HD microglia pathology in this extensively characterized iPSC line complements previous studies on Q109 iPSC-derived neurons^16,40^ and astrocytes^41,42^, and can help understand common and cell-type specific HD phenotypes. Although the average *HTT* CAG repeat length of HD patients is 41–45, research suggests that somatic instability of the CAG repeat tract explains clinical outcomes better than the length of the inherited allele^43,44^.

The influence of genetic background on disease phenotype can be significant. Therefore, the use of control iPSC lines that are genetically identical, besides CAG repeat length, is crucial to increase confidence in candidate disease phenotypes and mechanisms. Isogenic HD iPSC and ESC have been generated through a number of different methods, yet the piggyBac transposon was chosen for its ability to integrate large amounts of cargo and seamlessly excise vector sequences, leaving no ‘scars’ which have been found to influence expression of the gene it integrates into. We report a targeting efficiency of 3% similar to previous reports of genetic correction at the *HTT* locus (4.7–6% targeting efficiencies)^12,45^. Q22 iPSC maintained pluripotency and a normal karyotype and no new CNVs were identified in the corrected clones that were not present in the parent line, suggesting that these CNVs are not an artefact of cell culture, but likely existed in the patient. Though no off-target effects were observed, exome sequencing unsurprisingly revealed a number of non-synonymous SNPs between clones which likely arose through culture and illustrates the need for phenotypic assessment in multiple corrected iPSC clones.

Consistent with recent differentiation of HD hESC lines^19^, Q109 and Q60 iPSC were not compromised in their ability to differentiate into iPSC-microglia. However, HD MPC did show delayed adhesion and spreading on fibronectin substrate. Impaired adhesion and interaction with ECM is an established phenotype of HD neural progenitors^40^ and differential expression of ECM and integrin interacting genes are common features of HD RNA sequencing datasets^46^. This phenotype will therefore warrant further investigation particularly since altered ECM interaction is likely to impact the kinetics of immune cell behavior and function.

To investigate whether m*HTT* expression confers any cell-autonomous disease phenotypes in iPSC-microglia we performed a screen of cell and molecular phenotypes relevant to microglial function^28^. Using a cytokine array we first found that basal secretion of IL-6 was significantly increased in unstimulated Q109 compared to Q22 iPSC-microglia, as well as Q60 compared to Q33 iPSC-microglia. In addition, basal secretion of IL-8 was significantly increased in unstimulated Q109 compared to Q22 iPSC-microglia. Elevated levels of the pro-inflammatory cytokines IL-6, IL-8, IL-1β and TNF have been detected both centrally (in striatum and cerebrospinal fluid) and peripherally (in plasma) in HD patients^47–49^. Several PET studies, using the ligand translocator protein (TSPO), that is selectively expressed by activated microglia, have demonstrated that microglia activation correlates with disease severity in HD patients and represents one of the earliest clinical biomarkers of HD^5,6^. Activation of microglia is evident in pre-symptomatic HD gene carriers and can be detected up to 15 years before predicted age of onset^8^. Taken together, elevated basal microglia activity in the HD cell models and pre-manifest clinical data support the possibility of an early, cell-autonomous activation of microglia in early stages of the disease.

Following HD disease onset, and through later stages of disease progression, microglia function within an increasing neuroinflammatory microenvironment. We therefore also measured cytokine release after pro-inflammatory stimulation with LPS and IFN-γ and found that both Q109 and Q22 iPSC-microglia showed similar significant increases in IL-8 secretion. The secretion of IL-6 was also increased, although levels were highly variable. Previous studies with primary mouse microglia from the HD R6/2 transgenic model found that pro-inflammatory stimulation increased levels of IL-1β, IL-6, and TNF compared to control microglia^47,50^. Furthermore, microglia differentiated from hESC that contain 81 *HTT* CAG repeats exhibited a hyper-reactive response to stimulation with a high concentration of LPS and IFN-γ by showing an increased production of the pro-inflammatory cytokines IL-6 and TNF^19^. Such a hyper-reactive response was not detected in Q109 compared to Q22 iPSC-microglia, potentially because they reached a similar maximum cytokine secretion after stimulation with LPS and IFN-γ for 24 h.

m*HTT* expression induced further dysfunction in Q109 and Q60 iPSC-microglia. In contrast to a previous study that failed to detect an effect of m*HTT* on phagocytosis in HD ESC-derived microglia^19^, the Q109 iPSC-microglia showed a decreased capacity to phagocytose *E. coli* and to endocytose transferrin from the extracellular space, and the Q60 iPSC-microglia showed a decreased endocytosis of transferrin, but not phagocytosis of *E. coli*. The different results might be due to the cellular models used (ESC vs iPSC), as well as different culture conditions (e.g. the use of ACM) and differentiation protocols. Further studies on HD microglia phagocytosis/endocytosis would be of great benefit to better understand HD microglia pathology.

It is of interest that the differential uptake of *E. coli*, zymosan, dextran or transferrin by HD and control iPSC-microglia was cargo-specific and may implicate deficits in the different uptake mechanisms of extracellular material employed by phagocytosis and endocytosis. For example, the known role of the mHTT protein in clathrin-mediated endocytosis^51^ is consistent with the reduced uptake of transferrin seen in Q109 and Q60 iPSC-microglia. In contrast, *E. coli* and zymosan are phagocytosed by TLR4 and TLR2 dependent mechanisms respectively^52^. The phenotype of reduced *E. coli* phagocytosis may be consistent with a recent study that reported effects of mHTT on TLR4 signaling in mast cells^53^, and a genetic study that identified SNPs in the TLR4 and TREM2 genes as potential genetic modifiers of HD disease progression. Future studies that investigate mHTT protein interaction with TLR signaling in Q109 and Q20 cells and other models may shed further light on microglial phenotypes identified here.

In order to study the endosomal pathway, early and late endosome numbers were investigated. No differences in early RAB5 stained endosomes, but a significant decrease in late RAB7 stained endosomes was detected in Q109 compared to Q22 iPSC-microglia. Multiple proteins that interact with HTT are implicated in vesicle trafficking. The HTT-associated protein 40 (HAP40) has been identified as an effector of RAB5 as HAP40 mediates the recruitment of HTT by RAB5 onto early endosomes^54^. The huntingtin interacting protein (HIP1) is an endocytic protein whose structural integrity is crucial for maintenance of normal vesicle size^55^. This might indicate that m*HTT* expression directly influences the endosomal pathway in HD microglia.

Some of the key findings from the Q109/Q22 iPSC-microglia comparisons were replicated in Q60/Q33 iPSC-microglia. This pair of iPSC lines is not isogenic but were derived from affected and non-affected siblings and consequently share parental genetic backgrounds^40^. *HTT* CAG repeat lengths of 40–45 are most common in HD patients, and CAG repeat lengths above 60 are seen in ‘juvenile-onset HD’ patients with onset of symptoms before the age of 21^56^, however there is a growing notion that pathogenic forms of mHTT arise by massive somatic CAG repeat expansion prior to disease onset and throughout disease progression^44^. Using iPSC models with a high CAG repeat length, may be representative of cells expressing expanded pathogenic m*HTT* and has the advantage that these cells potentially exhibit more severe phenotypes and highlight changes in cellular pathology more clearly. Multiple papers have shown dose-dependent effects of the HTT CAG repeat length on phenotypes in iPSC-derived brain cells, including neurons and microglia^10,17, 19, 35^. Therefore, the isogenic Q109/Q20 lines provide powerful models to investigate novel HD microglia phenotypes. Results from the Q60/Q33 iPSC-microglia show that cytokine secretion and endocytosis are also affected in Q60 iPSC-microglia, indicating that this phenotype is likely to be induced by m*HTT* expression.

In summary, here we conducted a phenotypic screen of HD and isogenic control iPSC-derived microglial monocultures and provide evidence that m*HTT* expression causes cell-autonomous microglia dysfunction, notably to increase basal levels of pro-inflammatory cytokine secretion. This might help to understand why microglia activation can be detected in pre-symptomatic HD carriers. Furthermore, impacts on endocytosis and phagocytosis might affect efficient clearance of dying neurons during disease progression, which could in turn lead to further microglia activation. Notwithstanding acknowledged caveats of iPSC disease modelling such as loss of age-associated epigenetic changes that take place in patients^57^, future work using the Q109/Q22 isogenic lines in microglia/neuronal/astrocyte co-cultures will enable modelling of cell–cell interactions to address the precise contribution of microglia to HD pathology.

## Material and methods

### Cell lines and culture

The human induced pluripotent stem cell lines used in this study, CS109iHD-109n1, CS109iHD-109n4 and CS109iHD-109n5 (three clones, here combinedly named Q109), CS21iHD60n5 (Q60) and CS83iCTR-33n1 (Q33), were previously generated from human fibroblasts^35^. iPSC were cultured on Matrigel-coated plates (Corning) in Essential 8 Flex medium (Life Technologies) under standard culturing conditions (37 °C, 5% CO_2_).

### Molecular cloning of donor HDR plasmid

To generate the piggyBac targeting construct a 1.7 kbp 5’ homology sequence upstream of *HTT* and a 3’ homology arm containing human exon 1 with 22 CAG repeats were cloned into PB Multivector SGK-004 (Transposagen) following digestion with NotI and BsiWI, and NsiI and AscI, respectively. Primers 3’ homology arm-NsiI, 3’ homology arm- AscI, 5’ homology arm- NotI, 5’ homology arm- BsiWI amplifying the ligation sites were generated (Supplementary Table S4) and amplicons Sanger sequenced to confirm correct homology arm insertion.

### Generation of Q22 cell lines

A guide RNA (crRNA) targeting exon 1 of *HTT* (5'-GCCTCCGGGGACTGCCGTGCCGG-3') was generated (IDT). The crRNA was duplexed with a universal tracrRNA (IDT) (95 °C, 2 min) and then incubated with Alt-R S.p. Cas9 Nuclease 3NLS (IDT) (RT, 20 min) to form a ribonucleotide protein (RNP) complex. iPSC were dissociated with Accutase, and 1 × 10^6^ cells were nucleofected with 4 μg gRNA, 4 μg Cas9 and 1 μg PB donor HDR plasmid using the 4D-Nucleofector and P3 Primary Cell 4D- Nucleofector X Kit, with the program CA137 (Lonza). Immediately after transfection cells were replated into warmed E8 Flex medium, supplemented with 10 µM Y-27632 and 50 µM SCR7, a non-homologous end joining inhibitor. After a recovery period of 48 h in E8 Flex medium, cells were started on selection with conditioned medium containing 50 μg/mL geneticin (G418) for 10–13 days. Resistant colonies were picked into individual wells of a 96-well plate for screening and subsequent expansion. Screening was performed via PCR using primers amplifying the CAG repeat in *HTT* exon 1 (Supplementary Table S4).

To remove the selection cassette, targeted iPSC were electroporated with 1 μg PB Excision-Only Transposase Vector (Cambridge Biosciences). 48 h after nucleofection, cells were placed on selection with 1 μM ganciclovir for 5–7 days. Surviving colonies were picked and expanded for PCR screening. Primers amplifying exon 1 of *HTT* and sequencing primers were used for Sanger Sequencing to confirm successful genome-editing (Supplementary Table S4). Primers amplifying a region of the piggyBac donor vector (TK-NEO-cassette) were used to confirm the absence of the vector from the whole genome.

### *HTT* CAG repeat sizing with fragment analysis

DNA was extracted using the QIAamp DNA Mini Kit (QIAGEN), following manufacturer’s instructions. A fluorescently labelled forward primer and reverse primer, HTT-exon1 (Supplementary Table S1), were used to PCR amplify the CAG repeat region of *HTT*. Cycling conditions were as follows: initial denaturation at 94 °C for 90 s, followed by 35 cycles of 94 °C for 30 s, 65 °C for 30 s and 72 °C for 90 s and a final elongation at 72 °C for 10 min. PCR products underwent capillary electrophoresis using GeneScan LIZ600 dye Size Standard (Applied Biosystems), on the G3130xL Genetic Analyser (Applied Biosystems). Files were analyzed using GeneMapper (Applied Biosystems), Fragman^58^ and AutoGenescan (https://github.com/BranduffMcli/AutoGenescan). Quantification was performed with a 10% peak height threshold applied^59^.

### Off-target analysis

To predict potential off-target effects, guide sequences were analysed using CRISPOR^21^. The top 10 loci were selected, and flanking primers were designed for PCR amplification and Sanger sequencing. Off-target primers are listed in (Supplementary Table S1).

### SNP-based human microarray

Genomic DNA was extracted using QIAamp DNA Mini Kit (QIAGEN) and 200 ng (50 ng/µL) was required for genotyping. Samples were genotyped on the Infinium PsychArray-24 Kit (Illumina) or the Infinium Global Screening Array-24 (Illumina) and scanned using the iScan System (Illumina). Digital karyotypes were exported from Genome Studio (Illumina) and analyzed using PennCNV^60^. Sample level quality control was applied based on the standard deviation of Log R ratio set at 0.3, minimum SNP number of 10 and minimum region size of 100,000 bp.

### Whole exome sequencing

Exome sequencing was performed as described in detail in McAllister et al.^61^. Briefly, exome libraries were produced using TruSeq Rapid Exome Library Prep Kits (Illumina), according to manufacturer’s instructions. Libraries were sequenced on a HiSeq 4000 platform, and reads were aligned using the Burrows-Wheeler Aligner (BWA)^62^ to the GRCh37 (hg19) human reference genome. Variants were called using a standard Genome Analysis Toolkit (GATK) v3 pipeline^63^ (https://gatk.broadinstitute.org/hc/en-us), creating variant calling files (VCFs). Only variants which pass variant quality score recalibration (VQSR) were considered in analyses. Additional quality control measures were also employed, filtering variant calls < 10 reads or genotyping quality < 30. Variant annotation used a custom Hail pipeline (v0.1; https://github.com/hail-is/hail) which annotated variants using the variant effect predictor tool (VEP v95^64^), gnomAD (v 2.1.1^65^) and dbNSFP (v4.0b2^66^).

### Protein extraction and Western Blot

iPSC were washed once with cold DPBS and lysed with RIPA buffer (Sigma-Aldrich) containing cOmplete, EDTA-free Protease Inhibitor (Merck). Protein lysates were then denatured at 70 °C for 10 min in 1 × NuPAGE LDS Sample Buffer (Life Technologies) and 1 × NuPage Sample reducing Agent (Invitrogen). 15 µg of total protein extract per sample was separated on NuPAGE 3–8% Tris–Acetate gels (Invitrogen) alongside PageRuler Plus Prestained Protein Ladder (Thermo Scientific) using NuPAGE Tris–Acetate SDS Running Buffer (Invitrogen) containing NuPage Antioxidant (Invitrogen) at 150 V for 90 min. Gels were then transferred to nitrocellulose membrane, 0.45 µm (Bio-Rad) using NuPAGE Transfer Buffer (Invitrogen) at 135 V for 105 min. The membrane was blocked in 5% milk in PBS-T and incubated overnight at 4 °C with anti-HTT (MAB2166 clone 1HU-4C8, Sigma-Aldrich, mouse monoclonal, 1:1000) and anti-β-tubulin (ab6046, Abcam, rabbit polyclonal, 1:2000). IRDye 800CW Donkey anti-Mouse IgG (LI-COR Biosciences, 926-32212, 1:15,000) and Goat anti-Rabbit IgG Alexa Fluor 680 (Invitrogen, A27042, 1:5000) were used as secondary antibodies. Immunoblots were visualized with the Odyssey CLx Imaging System (LI-COR).

### Generation of iPSC-derived microglia

iPSC were differentiated to iPSC-microglia using a protocol adapted from Haenseler et al.^23^. Briefly, iPSC were treated with 10 μM Y-27632 1 h before dissociation using Accutase. iPSC were resuspended in Essential 8 Flex medium containing 10 μM Y-27632, 50 ng/mL BMP4, 50 ng/mL VEGF121 and 50 ng/mL SCF and plated at a final concentration of 10^4^ cells/100 μL in ultra-low attachment 96-well plates (Corning Costar). Cells were incubated for 4 days with minimal disturbance, including a 50% medium change on day 2. On day 4, embryoid bodies were collected and transferred to 6-well plates changing medium to X-VIVO15 (Lonza) containing 1% Pen/Strep, 1% Glutamax, 50 nM β-Mercaptoethanol, 50 ng/mL M-CSF and 50 ng/mL IL-3. Two-thirds of the medium was changed every 5–7 days. After approximately 25 days, microglia precursor cells (MPC) were harvested once per week for microglia differentiation. Non-adherent MPC were collected and cultured in astrocyte-conditioned media for 12–14 days with a full medium change after 1 week. iPSC-derived astrocytes were differentiated as previously described^67^. ACM was derived by conditioning Advanced DMEM/F-12 (Life Technologies) for 48 h. The collected media were pooled, sterile filtered and frozen at − 80 °C until further used. Each batch was tested with a CCL2 ELISA (Biotechne), which was used as a housekeeping reference for the amount of protein / sample. All batches were diluted down to a CCL2 concentration of 1 ng/mL.

### Flow cytometry for surface proteins

Flow cytometry was performed to analyze the myeloid cell surface markers CD11b, CD14 and CD45 on the non-adherent MPC collected after day 25 of differentiation. MPC were blocked using 1% FBS in PBS for 30 min at room temperature and subsequently stained with either the antibody or isotype control in 1% FBS in PBS for 30 min on ice. Following antibodies were used: CD11b-APC (301309, Biolegend), APC Mouse IgG1 (400119, Biolegend), CD14-APC (17-0149-42, Invitrogen), APC Mouse IgG1 (17-4714-42, Invitrogen), CD45-FITC (304005, Biolegend), FITC Mouse IgG1 (400307, Biolegend). Cells were washed with 1% FBS in PBS for 3 times and then fixed for 10 min using 4% paraformaldehyde. Cells were washed again and measured on the LSR Fortessa (BD Biosciences). Data were analyzed using FlowJo software (BD Biosciences).

### Pro-inflammatory stimulation

iPSC-microglia were stimulated with 20 ng/mL IFN-γ and 20 ng/mL LPS for 24 h prior to experimental procedures.

### Cell spreading assay

MPC were seeded at 1 × 10^4^ cell/well in optically clear bottom half area 96-well plates (Greiner). Cell spreading was analyzed by a time course, where the cells were fixed 15 min, 60 min and 120 min after plating onto 5 μg/mL fibronectin. MPC were fixed for 10 min using 4% PFA, followed by 3 washes with PBS. MPC were permeabilized for 3 min with 0.1% Triton X-100 in PBS. After 2 washes with PBS, cells were stained with Phalloidin-iFluor 555 (ab176756, Abcam) and Hoechst 33342 (H3570, Invitrogen) for 90 min in 1% BSA in PBS. Cells were washed 2 times with PBS and imaged at the Opera Phenix High-Content Screening System (Perkin Elmer) with 40 × water objective. Harmony software was used for image analysis and “Cell Roundness” chosen as the output measure. A roundness score of 1 represents a circle.

### Analysis of iPSC-microglia morphology

MPC were seeded at 1 × 10^4^ cell/well in optically clear bottom half-area well 96-well plates (Greiner) and differentiated for 14 days. Q109 and Q22 iPSC-microglia were fixed for 10 min using 4% PFA, followed by 3 washes with PBS. Cells were permeabilized for 3 min with 0.1% Triton X-100 in PBS. After 2 washes with PBS, cells were stained with Phalloidin-iFluor 555 and Hoechst 33342 for 90 min in 1% BSA in PBS. Cells were washed 2 times with PBS. Q60 and Q33 iPSC-microglia were stained live with CellTracker Deep Red (Invitrogen) for 30 min, followed by one wash with Live Cell Imaging Solution (Invitrogen). iPSC-microglia were imaged at the Opera Phenix High-Content Screening System (Perkin Elmer) with 40 × water objective. The Harmony software was used for image analysis and “Cell Area” and “Cell Roundness” chosen as the output measure.

### Human inflammatory cytokine assay

MPC were seeded at 2 × 10^4^ cell/well in 96-well plates (Greiner) and differentiated for 14 days. Q109 and Q22 iPSC-microglia secreted cytokines were quantified using the flow-cytometry based BD Cytometric Bead Array (CBA) Human Inflammatory Cytokine Kit. iPSC-microglia supernatant was collected 12 h after a full medium change. For each sample, supernatant from 3 wells were pooled. Replicates were collected on different days and run as distinct experiments. Experiments were conducted according to manufacturer’s instructions and run on the BD LSR Fortessa flow cytometer. Samples were analyzed using FlowJo software and the median PE value was used. Standard curves were generated in Excel and cytokine concentrations calculated accordingly. Concentrations were normalized to unstimulated Q22 iPSC-microglia.

### ELISA

MPC were seeded at 2 × 10^4^ cell/well in 96-well plates (Greiner) and differentiated for 14 days. Q60 and Q33 iPSC-microglia secreted cytokines were quantified using the IL-8/IL-6 Human Uncoated ELISA Kit (Invitrogen), in accordance with the manufacturer’s protocol. Chemiluminescent signal was measured on a plate reader at 450 nm and standard curves were generated in Excel.

### Phagocytosis and endocytosis assays

MPC were seeded at 1 × 10^4^ cell/well in optically clear bottom half-area well 96-well plates (Greiner) and differentiated for 14 days. To study phagocytosis 10 μg/mL pHrodo Red *E. coli* BioParticles (Invitrogen) and 50 μg/mL pHrodo Red Zymosan Bioparticles (Invitrogen) were used. To inhibit the phagocytosis, 30 μM cytochalasin D (CytoD; Bio-Techne) was added 1 h prior and during the assay. To study endocytosis 50 μg/mL pHrodo Red Transferrin (Invitrogen) and 5 μg/mL pHrodo Green Dextran (Invitrogen) were used. To inhibit endocytosis, 30 μM dynasore (Bio-Techne) was added 1 h prior and during the assay. iPSC-microglia were stained with CellTracker Deep Red and the nuclear stain NucBlue (Invitrogen) 30 min at 37 °C prior to the assay. Medium was changed to live cell imaging solution and pHrodo particles added. Plates were directly transferred to the Opera Phenix High-Content Screening System (Perkin Elmer) with temperature set to 37 °C and CO_2_ to 5%. Images were taken every 15 or 20 min for 4 h with 40 × water objective. The Harmony software was used for image analysis and “Total Spot Area” chosen as the output measure.

### Immunocytochemistry

Cells were fixed with 4% PFA for 10 min, followed by 3 washed with PBS. Fixed iPSC and iPSC-microglia were incubated in blocking buffer (3% BSA, 3% serum in PBS) for 1 h at room temperature, followed by incubation in primary antibody (diluted in blocking buffer) over night at 4 °C. Cells were washed 3 times for 5 min with PBS, followed by incubation in the secondary antibody (diluted in blocking buffer) for 1 h at room temperature. Antibodies are listed in Supplementary Table S5. Cells were again washed 3 times for 5 min with PBS and incubated with Hoechst 33342 (1:5000 in blocking buffer) for 10 min at room temperature. Cells were washed 3 times for 5 min with PBS and stored at 4 °C until imaged. Images were taken at the Opera Phenix High Content Imaging System (Perkin Elmer) or Cell Observer spinning disc confocal (Zeiss).

### Calcium imaging

MPC were seeded at 1 × 10^4^ cell/well in optically clear bottom half-area well 96-well plates (Greiner) and differentiated for 14 days. iPSC-microglia were stained with 2 μM Fluo8 (Abcam) for 1 h at RT and then replaced with Live Cell Imaging Solution. Intracellular calcium changes were induced by 1 μM Ionomycin (Merck), 50 μM ATP (Sigma) and 50 μM ADP (Sigma). Calcium imaging was conducted in the FLIPR Penta High-Throughput Cellular Screening System (Molecular Devices). Fluorescent changes were recorded every second and an output per well generated. Raw fluorescent intensity data were used to calculate the increase in intracellular calcium after stimulation compared to baseline. Fluorescent data were normalized by cell count generated using the Opera Phenix High Content Screening System (Perkin Elmer) directly after calcium imaging.

### Seahorse XF cell mito stress test

The Seahorse XF Cell Mito Stress Test Kit was used according to manufacturer’s protocol. MPC were seeded at 4 × 10^4^ cell/well in PDL-coated XF Cell Culture microplates and differentiated to iPSC-microglia for 14 days. The Seahorse XF96 sensor cartridge was hydrated overnight at 37 °C in a non-CO2 incubator using 200 μL Seahorse XF calibrant / well. Assay media were prepared freshly on the day of the experiment. Seahorse XF DMEM medium was supplemented with 1 mM pyruvate, 2 mM glutamine and 10 mM glucose. 175 μL assay medium was added to each well of the cell culture microplate and it was then incubated for 45 min at 37 °C in a non-CO_2_ incubator. Compounds were prepared at 10 × stock concentration in assay medium: 20 μM oligomycin, 20 μM carbonilcyanide p-triflouromethoxyphenylhydrazone (FCCP) and 5 μM rotenone and antimycin A. Compounds were then added to the hydrated sensor cartridge ports. The hydrated sensor cartridge and cell culture microplate were inserted into the Seahorse XFe96 instrument, and the Cell Mito Stress Test protocol run using the Wave software.

### DQ-BSA

MPC were seeded at 1 × 10^4^ cell/well in optically clear bottom half-area well 96-well plates (Greiner) and differentiated for 14 days. iPSC-microglia were incubated with 1 μM Bafilomycin (Bio-Techne), together with CellTracker Far Red and NuncBlue for 1 h at 37 °C. Cells were washed once and 10 μg/mL DQ-Green BSA (Invitrogen) was added for 2 h. The Opera Phenix High-Content Screening System with temperature set to 37 °C and CO_2_ to 5% was used to image the cells live with 40 × water objective. The Harmony software was used for image analysis and “Number of Spots per Cell” and “Total Spot Area” chosen as the output measure.

### Statistics

Statistical analysis as well as graph design was carried out using GraphPad Prism 8 (GraphPad Software). Unpaired t-test was used for comparison of two groups at a single timepoint. When data did not pass testing for normality, Mann–Whitney *U* test was chosen. Two-way ANOVA with Tukey's honest significance test was used when cell line and timepoint or cell line and stimulation were factors. Results are reported as mean values ± SEM.

### Supplementary Information

Supplementary Information.

## Data Availability

The data sets analyzed for this study are available from the corresponding authors on reasonable request.
