# Supplementary material for: Mutant huntingtin confers cell-autonomous phenotypes on Huntington’s disease iPSC-derived microglia
Source: Sci Rep. 2023 Nov 22;13:20477. doi: 10.1038/s41598-023-46852-z (PMC10665390; doi:10.1038/s41598-023-46852-z)
Supplement: Supplementary file 1 — Supplementary Information. [file 41598_2023_46852_MOESM1_ESM.docx]

# Supplementary Information

Mutant huntingtin confers cell-autonomous phenotypes on Huntington’s disease iPSC-derived microglia

Nina Stöberl ^1^*, Jasmine Donaldson ^2^, Caroline S. Binda ^2^, Branduff McAllister ^2^, Hazel Hall-Roberts ^3^, Lesley Jones ^2^, Thomas H. Massey ^2^ & Nicholas D. Allen ^1^*

1 School of Biosciences, Cardiff University, Cardiff, UK

2 Centre for Neuropsychiatric Genetics and Genomics, Division of Psychological Medicine and Clinical Neurosciences, Cardiff University, Cardiff, UK

3 UK Dementia Research Institute at Cardiff, Cardiff University, Cardiff, UK

* Corresponding authors: StoberlN@cardiff.ac.uk & AllenND@cardiff.ac.uk

**Supplementary Figure S1**: Validation of Q22 iPSC. (**a**) Sanger sequencing confirmed the PB HDR vector cassette sequence (upper) and excision of the piggyBac cassette from TTAA integration sites and introduction of silent PAM site mutations (GGG > CCG) (lower). (**b**) Sanger sequencing showed absence of expanded repeat in Q22 clones. (**c**) Electropherograms of fluorescent PCR across CAG repeat showed two WT alleles (21 CAG, 22 CAG) present in targeted Q22 clones.

**Supplementary Figure S2:** Characterisation of iPSC-microglia differentiation. (**a**) Overview of iPSC-microglia differentiation protocol adapted from Haenseler et al., 2017. The addition of BMP-4, SCF and VEGF to iPSC led to the development of embryoid bodies biased to mesoderm and haematopoietic differentiation. Further addition of the factors M-CSF and IL-3 guided the production of non-adherent myeloid cells in the culture supernatant. In a second step these non-adherent microglia precursor cells (MPC) were harvested and differentiated to a microglial phenotype by the addition of astrocyte conditioned medium. Scale bars = 500 μm for iPSC, 200 μm for embryoid bodies, 200 μm for factories, 100 μm for MPC and iPSC-microglia. (**b**) MPC were stained for myeloid surface markers CD11b, CD14, and CD45, and were investigated by flow cytometry. No differences were detected between Q22, Q33, Q60 and Q109 MPC. Data are presented as mean ± SEM. n = 2-3 clones, in two independent experiments. Statistical analysis was performed using unpaired Student´s t-test.

**Supplementary Figure S3**: Phagocytosis and endocytosis impairment is cargo-specific. (**a**) Phagocytosis was assessed via the uptake of pHrodo-conjugated zymosan. Q109 and Q22 iPSC-microglia were imaged every 15 minutes for 4 hours. Uptake of zymosan was inhibited by the addition of 30 μM cytochalasin D (CytoD) for 1 hour prior and during phagocytosis. Total spot area of phagocytosed zymosan after 4 hours was not significantly between Q109 and Q22 control iPSC-microglia. (**b**) Endocytosis was assessed via the uptake of pHrodo-conjugated dextran. Q109 and Q22 iPSC-microglia were imaged every 15 min for 4 hours. Uptake of dextran was inhibited by the addition of 30 μM dynasore for 1 hour prior and during endocytosis. Total Spot Area of dextran was not significantly different between Q109 and Q22 control iPSC-microglia. Data are expressed as mean ± SEM. n = 3 independent experimental repeats with 2-3 clones. Statistical analysis was performed using unpaired Student´s t-test.

**Supplementary Figure S4**: Q109 iPSC-microglia showed impairment in calcium responses, but not in mitochondrial respiration and protease activity. (**a**) General calcium handling using ionomycin and response to the purines ATP and ADP was measured using Fluo-8 AM and the FLIPR Penta High-Throughput Cellular Screening System. Increase in intracellular calcium as a response to 1 μM ionomycin was significantly decreased in Q109 compared to Q22 iPSC-microglia. ATP and ADP increase intracellular calcium concentrations in iPSC-microglia by specific ionotropic and metabotropic purinergic receptors on the microglial membrane. Q109 iPSC-microglia showed significantly lower calcium responses to 50 μM ATP, but not to 100 μM ADP, compared to Q22 iPSC-microglia. (**b**) Mitochondrial respiration was investigated with the Seahorse XF Cell Mito Stress Test. No differences in oxygen consumption rate (OCR) were detected between Q109 and Q22 iPSC-microglia. (**c**) Protease activity was investigated using DQ-BSA, together with the cytosolic dye CellTracker and nuclear stain Hoechst. Number of DQ-BSA puncta / cell and total DQ-BSA area were investigated. The V-ATPase inhibitor bafilomycin A1 was used to inhibit the degradation of DQ-BSA. No differences were detected between Q109 and Q22 iPSC-microglia. Bafilomycin A1 successfully inhibited proteolytic activity. Scale bar = 25 μm. Data are expressed as mean ± SEM. n = 3 biological replicates repeated in 3 independent experiments. Statistical analysis was performed using two-way ANOVA and Tukey's honest significance test. ** p < 0.01, *** p < 0.001.

**Supplementary Figure S5**: Uncropped images supporting Figure 1. (**a**) Uncropped PCR gel used in Figure 1d. (**b**) Uncropped PCR gel used in Figure 1e. (**c**) Uncropped PCR gels used in Figure 1g. (**d**) Uncropped Western Blot used in Figure 1i. (**e**) additional Western Blot replicates, similar to (d).

**Supplementary Table S1**: Potential CRISPR off-targets in Q22 iPSC

| **Off-target site** | **Homologous sequence (mismatches in red)** |
| --- | --- |
| *chr4:3038378* | GCCTCTGAGGACAGCAGTGCCGG |
| *chr10:95973998* | GCATCTGGGGACTGCAGTACGGG |
| *chr14:65238946* | GCCTCAGAGGACTGCCATGAGGG |
| *chrX:153251031* | TCCTCCGTGGACTGCAGAGCTGG |
| *chr15:74490998* | GCCTCCATGGACAGCCATGCAGG |
| *chr22:47647181* | GCCCCAGGGGACTGCAGAGCGGG |
| *chr6:78176493* | GCCGCTGGGGACTGCAGAGCTGG |
| *chr11:15132157* | GCCCCATGGGACTGCCATGCTGG |
| *chr17:76462925* | GCCTACTGGGACTGCAGTGCTGG |
| *chr11:65668058* | GCCTCCGGGGAACGCCGAGCCGG |

**Supplementary Table S2**: Single nucleotide variants between cell lines identified by whole exome sequencing

| **Variant** | **Gene** | **Q109** | **Q22-1** | **Q22-2** | **Q22-3** | **Q109-1** |
| --- | --- | --- | --- | --- | --- | --- |
| 1:2560818:CCAG:C | *MMEL1* | ./. | ./. | 0/0 | 0/1 | 0/0 |
| 1:9098943:CCAG:C | *SLC2A5* | 0/0 | 0/1 | 0/0 | 0/0 | 0/0 |
| 1:89449508:AT:A | *RBMXL1* | 0/1 | ./. | ./. | 0/0 | ./. |
| 1:104076466:G:GA | *RNPC3* | 0/0 | 0/1 | ./. | ./. | ./. |
| 1:208063100:G:GAA | *CD34* | ./. | 0/1 | 0/0 | ./. | ./. |
| 1:248524937:ATGGGACTCTTCAGACAATCCAAACATCCAATGGCCAATATCACCTGGATGGCCAACCACACTGGATGGTCGGATTTCATCCTGT:A | *OR2T4* | 0/1 | 0/0 | 0/0 | 0/0 | 0/0 |
| 2:153476066:G:GCCA | *FMNL2* | ./. | 0/1 | 0/1 | 1/1 | ./. |
| 2:170632960:C:CA | *KLHL23* | 0/1 | 0/0 | ./. | ./. | ./. |
| 2:178988919:CA:C | *RBM45* | ./. | 0/0 | 0/0 | 0/1 | ./. |
| 2:203651475:A:AT | *ICA1L* | 0/1 | 0/0 | ./. | ./. | 0/1 |
| 2:215940283:G:GA | *ABCA12* | 0/1 | 0/0 | ./. | ./. | ./. |
| 2:231334495:TA:T | *SP100* | 0/1 | ./. | ./. | 0/0 | ./. |
| 2:241696840:ATCC:A | *KIF1A* | ./. | 0/0 | ./. | 0/1 | ./. |
| 3:42700746:GGGA:G | *ZBTB47* | 0/0 | 0/1 | ./. | ./. | ./. |
| 3:75786737:A:T | *ZNF717* | 1/1 | ./. | ./. | 0/1 | ./. |
| 3:112329033:G:GA | *CCDC80* | 0/1 | ./. | ./. | 0/0 | ./. |
| 3:195506270:GACCTGTGGATAATGAGGAAGCATTGGTGACAGGAAGAGGGGTGGTGTC:G | *MUC4* | 0/0 | 0/0 | 1/1 | 1/1 | ./. |
| 3:195506290:GCA:G | *MUC4* | 0/1 | ./. | 0/0 | 0/0 | 0/0 |
| 3:195506293:T:TGC | *MUC4* | 0/1 | ./. | 0/0 | 0/0 | 0/0 |
| 3:195510749:C:A | *MUC4* | ./. | 1/1 | ./. | 1/1 | 0/1 |
| 4:1019054:CCA:C | *FGFRL1* | ./. | 0/1 | ./. | 0/0 | 0/0 |
| 4:3443797:CCTG:C | *HGFAC* | ./. | ./. | 0/0 | 0/1 | ./. |
| 4:57180576:C:CGGAGCGGAGGGAGCGGAG | *CRACD* | 0/1 | ./. | 0/1 | ./. | 0/0 |
| 4:74347531:GT:G | *AFM* | 0/0 | 0/0 | 0/0 | 0/1 | 0/0 |
| 4:88929173:CGAG:C | *PKD2* | ./. | 0/0 | 0/1 | ./. | ./. |
| 4:140811083:GC:G | *MAML3* | 1/1 | 1/1 | 0/0 | ./. | 0/0 |
| 4:140811085:TGCTGCTGCTGC:T | *MAML3* | 1/1 | 1/1 | 0/0 | ./. | 0/0 |
| 5:113822457:TA:T | *KCNN2* | ./. | ./. | 0/1 | 0/1 | 0/0 |
| 5:171201605:G:GC | NA | ./. | ./. | 0/1 | ./. | 0/0 |
| 6:32606717:TAGAG:T | *HLA-DQA1* | 0/0 | 0/0 | ./. | 0/0 | 0/1 |
| 6:32606730:T:TG | *HLA-DQA1* | 0/0 | 0/0 | ./. | 0/0 | 0/1 |
| 6:32629141:C:T | *HLA-DQB1* | 0/1 | 0/1 | 0/1 | 0/0 | ./. |
| 6:32725559:C:T | *HLA-DQB2* | ./. | ./. | 0/1 | 0/0 | ./. |
| 6:32725567:C:T | *HLA-DQB2* | ./. | ./. | 0/1 | 0/0 | ./. |
| 6:57398186:G:GA | *PRIM2* | 0/0 | 0/1 | ./. | ./. | ./. |
| 6:100006295:T:TA | *CCNC* | 0/1 | 0/0 | ./. | ./. | 0/0 |
| 6:160505207:A:G | *IGF2R* | 0/1 | 0/1 | 0/1 | 1/1 | 0/1 |
| 7:100547299:G:T | NA | 0/1 | 0/1 | ./. | 0/0 | ./. |
| 7:100552358:C:G | *MUC3A* | 0/1 | 0/0 | 0/1 | 0/1 | 0/1 |
| 7:100552371:C:G | *MUC3A* | 0/1 | 0/0 | 0/1 | 0/1 | 0/1 |
| 7:100612955:A:ACTG | *MUC12* | 0/1 | 0/1 | 0/0 | 0/1 | 0/1 |
| 7:100616336:G:A | *MUC12* | ./. | 0/0 | ./. | 0/1 | ./. |
| 7:100616338:A:G | *MUC12* | ./. | 0/0 | ./. | 0/1 | ./. |
| 7:100679136:T:C | *MUC17* | 0/1 | 1/1 | ./. | ./. | ./. |
| 8:52733231:G:A | *PCMTD1* | ./. | ./. | ./. | 0/0 | 0/1 |
| 8:87951852:C:A | *CNBD1* | 0/0 | ./. | ./. | 0/0 | 0/1 |
| 8:101724994:C:CA | *PABPC1* | ./. | 0/1 | 0/1 | 0/0 | ./. |
| 9:35906347:CCTG:C | *HRCT1* | 0/1 | ./. | ./. | 0/0 | ./. |
| 10:51573343:C:CA | NA | ./. | 0/0 | ./. | ./. | 0/1 |
| 11:550204:C:T | *LRRC56* | 0/0 | 0/0 | 0/1 | 0/0 | 0/0 |
| 11:1606120:ACCACAGCCACCCTTGGATCCCCCACAAGAG:A | *KRTAP5-1* | 0/1 | 0/1 | 0/1 | 0/1 | 0/0 |
| 11:1619172:ACCCCCACAGGAGCCACAGCCCCCCTTGGAG:A | *KRTAP5-2* | 0/1 | 0/0 | ./. | 0/1 | ./. |
| 11:1651094:A:AGGCTGTGGCTCCGGCTGTGGAGGCCTTGGCTCCGGCTGTGGG | *KRTAP5-5* | 0/0 | 0/1 | 0/0 | ./. | ./. |
| 11:6411935:TGCTGGC:T | *SMPD1* | 0/1 | 0/0 | ./. | 0/1 | 0/0 |
| 11:9462016:G:A | *IPO7* | 0/0 | 0/0 | 0/0 | 0/0 | 0/1 |
| 11:45241245:G:A | *PRDM11* | 0/1 | 1/1 | 0/1 | 0/1 | 0/0 |
| 11:104879686:CT:C | *CASP5* | ./. | ./. | 0/0 | ./. | 0/1 |
| 11:108357082:C:A | *POGLUT3* | 0/1 | 0/0 | 0/0 | 0/0 | 0/0 |
| 11:115080311:ATGG:A | *CADM1* | 0/0 | 0/0 | 0/0 | 0/1 | 0/0 |
| 12:18854540:ATCCTCCTCCTCCTCC:A | *PLCZ1* | 0/1 | 0/0 | ./. | 0/0 | 0/0 |
| 12:109998845:C:G | *MMAB* | 0/0 | 0/0 | 0/0 | 0/0 | 0/1 |
| 12:117289548:TA:T | *RNFT2* | ./. | 0/0 | 0/1 | ./. | 0/1 |
| 12:121678327:C:CTTT | *CAMKK2* | 0/1 | 0/1 | ./. | ./. | 0/0 |
| 12:121678327:C:CTTTT | *CAMKK2* | ./. | 0/0 | ./. | ./. | 0/1 |
| 12:125465269:ACTC:A | *DHX37* | 0/0 | 0/0 | 0/0 | ./. | 0/1 |
| 13:72440443:ACTG:A | NA | ./. | ./. | 0/1 | 0/0 | ./. |
| 14:23371255:G:GGGC | *RBM23* | 0/1 | ./. | 0/1 | ./. | 1/1 |
| 14:74943295:T:C | *NPC2* | 0/1 | 0/0 | 0/1 | 0/1 | 0/1 |
| 14:92537353:C:CG | *ATXN3* | 1/1 | 1/1 | 1/1 | 0/1 | 1/1 |
| 14:92537354:C:CTGCTGCTGCTGCTGCTG | *ATXN3* | 1/1 | 1/1 | 1/1 | 0/1 | 1/1 |
| 14:92537354:C:G | *ATXN3* | 0/0 | 0/0 | 0/0 | 0/1 | 0/0 |
| 15:82637079:C:T | NA | 1/1 | ./. | ./. | 1/1 | 0/1 |
| 17:7750177:T:TACCACC | *KDM6B* | ./. | 1/1 | 0/1 | 0/1 | ./. |
| 17:17697093:CCAG:C | *RAI1* | 0/0 | 0/0 | 0/0 | ./. | 0/1 |
| 17:30796174:C:G | *PSMD11* | 0/0 | 0/1 | 0/0 | 0/1 | 0/0 |
| 17:39411670:T:TACCTGCTGCAGGACC | *KRTAP9-9* | 0/1 | 0/0 | ./. | ./. | 0/0 |
| 18:9887388:GGAAGCCATCCAGCCCAAGGAGGGTGACATCCCCAAGTCCCCAGAA:G | *TXNDC2* | 0/0 | 0/0 | 1/1 | 0/0 | 0/0 |
| 18:14848820:C:T | *ANKRD30B* | 0/1 | 0/1 | 1/1 | 0/1 | 0/1 |
| 18:48331573:C:T | *MRO* | 0/0 | 0/0 | 0/0 | 0/0 | 0/1 |
| 19:501701:G:A | *MADCAM1* | 0/0 | ./. | ./. | 0/1 | ./. |
| 19:501701:G:GACACCACCTCCCCGGAGCCTCCCA | *MADCAM1* | 0/1 | ./. | ./. | 0/0 | ./. |
| 19:501719:T:C | *MADCAM1* | ./. | ./. | 0/1 | 0/0 | 0/1 |
| 19:8999443:T:C | *MUC16* | 0/0 | 0/0 | ./. | 0/1 | ./. |
| 19:9361740:CT:C | *OR7E24* | 0/0 | 0/1 | ./. | ./. | ./. |
| 19:10752069:C:CA | NA | ./. | 0/1 | 0/0 | 0/0 | ./. |
| 19:39760435:T:C | *IFNL2* | 0/1 | ./. | ./. | ./. | 0/0 |
| 19:41173892:CTGCTGT:C | *NUMBL* | 0/1 | 1/1 | 0/1 | ./. | 1/1 |
| 19:56539724:G:T | *NLRP5* | 0/0 | 0/0 | 0/1 | 0/0 | 0/0 |
| 21:47855916:C:T | *PCNT* | 0/1 | 0/1 | 0/1 | 0/1 | 0/0 |
| 22:22707527:C:T | *IGLV5-48* | 0/1 | 0/1 | ./. | 0/0 | ./. |
| X:139866459:CGTCTTCCAACAAAGGTAT:C | NA | 0/1 | ./. | ./. | 0/1 | 0/0 |
| X:139866477:T:C | NA | 0/0 | ./. | ./. | 0/0 | 0/1 |
| X:140993801:GCTCCTTCTCCTCCACTTTATTGAGTATTTTCCAGAGTTCCCCTGAGAGAACTCAGAGTACTTTTGAGGGTTTTGCCCAGTCTCCTCTCCAGATTCCTGTGAGCCCCTCCTCCTCCTCCACTTTACTGAGTCTTTTCCAGAGTTTCTCTGAGAGAACTCAGAGTACTTTTGAGGGTTTTGCCCAGTCTTCTCTCCAGATTCCTGTGAGCCC:G | *MAGEC1* | 0/0 | 0/0 | 0/0 | 0/0 | 0/1 |
| X:140993827:A:C | *MAGEC1* | ./. | 0/1 | ./. | ./. | 0/0 |
| X:153590793:G:T | *FLNA* | 0/1 | 0/1 | 0/1 | ./. | 0/0 |

**Supplementary Table S3**: CNV analysis of iPSC clones

| **CNV locus** | **Position** | **Status** | **Length (bp)** | **Gene** |
| --- | --- | --- | --- | --- |
| 2q22.1 | chr2:141448312-141932472 | Deletion | 484,161 | *LRP1B* |
| 6q23.3 | chr6:136303107-136376393 | Deletion | 73,287 | *PDE7B* |
| 12q14.2 | chr12:64011062-64116159 | Duplication | 105,097 | *DPY19L2* |
| 14q24.3 | chr14:77984540-78108110 | Deletion | 123,571 | *SPTLC2* |

**Supplementary Table S4**: List of primers

| **Name** | **Forward (F)/ Reverse (R)** | **Sequence (5’-3’)** |
| --- | --- | --- |
| Donor Vector construction | | |
| 3’ homology arm-NsiI | F | CTAAATGCACAGCGACGGAT |
|  | R | CTGAGGAAGCTGAGGAGGC |
| 3’ homology arm- AscI | F | GATGTGTCCCAGATGGCATT |
|  | R | CCATGATTACGCCAAGCTCG |
| 5’ homology arm- NotI | F | GTTGTAAAACGACGGCCAGT |
|  | R | GGGGCGTTTCTTTATGGGAG |
| 5’ homology arm- BsiWI | F | CCATTCATTGCCCCGGTG |
|  | R | TCCAGACTGCCTTGGGAAAA |
| Screening and HTT CAG repeat sizing | | |
| F1/ R1 | F | ATGAAGGCCTTCGAGTCCCTCAAGTCCTTC |
|  | R | GGCGGCTGAGGAAGCTGAGGA |
| F3/ R3 | F | CCCATTCATTGCCCCGGTGCTG |
|  | R | TGGGTTGCTGGGTCACTCTGTC |
| Clone screening | | |
| F2/ R2 | F | TCGCCTTCTTGACGAGTTCT |
|  | R | GCAGATACCGCACCGTATTG |
| LHA-junction | F | TACAGGCATGCACCACTACA |
|  | R | CATCAGAGCAGCCGATTGTC |
| RHA-junction | F | TACCCGAGCCGATGACTTAC |
|  | R | ATCAAGTGTCTGAAGCAGCC |
| Off-target | | |
| 1_OT_SNP | F | AAGTTTCTCAGGACTGCGGG |
|  | R | CATGCCCTTCTCCTTTCCGT |
| 2_OT_SNP | F | CTGCCCTTCCCAGAAGACTG |
|  | R | GTGTGCACCTGTCACTGGTA |
| 3_OT_SNP | F | GGCAGGGGTCTGAACAGTAG |
|  | R | GTGTCCAGGTACCTTCAGCC |
| 4_OT_SNP | F | ACAGCTTCCTGCATCCCTTC |
|  | R | AGGAAGAAGCCACGTGGAAG |
| 5_OT_SNP | F | ATGATCTCCCCTGGTCCCTC |
|  | R | AATGCAGCCTTGATGCCTCT |
| 6_OT_SNP | F | CTCACTGTAGCAGGGGCAAA |
|  | R | TGCCCATCTTGAAGCCACAT |
| 7_OT_SNP | F | GGAGCATGGGACGGAGAAAA |
|  | R | CCCTTCCGCTACTGATTCCC |
| 8_OT_SNP | F | CGAAGTCTCGGAACATGCCC |
|  | R | TCACTCCACGAAGCTTTGGG |
| 9_OT_SNP | F | CTGGGAACTTTCTGGCTCGT |
|  | R | CTCCACAGCACCTTCTCCAG |
| 10_OT_SNP | F | GGAGATTAAGCACTCCTCCCTC |
|  | R | GGTAGAGCAGAGCACGTGTT |

**Supplementary Table S5**: List of immunocytochemistry antibodies

| **Antibody** | **Host species** | **Concentration** | **Article number** | **Company** |
| --- | --- | --- | --- | --- |
| Primary antibodies | | | | |
| OCT4 | Mouse | 1:100 | SC5279 | Santa Cruz |
| IBA1 | Goat | 1:100 | AB5076 | Abcam |
| TMEM119 | Rabbit | 1:100 | AB185333 | Abcam |
| RAB5 | Rabbit | 1:1000 | AB218624 | Abcam |
| RAB7 | Mouse | 1:500 | AB50533 | Abcam |
| Secondary antibodies | | | | |
| 594 anti-goat | Chicken | 1:400 | A-21468 | Invitrogen |
| 488 anti-rabbit | Chicken | 1:400 | A-21441 | Invitrogen |
| 594 anti-rabbit | Goat | 1:400 | A-11012 | Invitrogen |
| 488 anti-mouse | Goat | 1:400 | A-11001 | Invitrogen |
